# African schistosomes in small mammal communities: Perspectives from a spatio-temporal survey in the vicinity of Lake Guiers, Senegal

**DOI:** 10.1371/journal.pntd.0012721

**Published:** 2024-12-23

**Authors:** Julien Kincaid-Smith, Boris Sègnito A. E. Savassi, Bruno Senghor, Christophe Diagne, Youssoupha Niang, Mamadou Kane, Caroline Tatard, Carine Brouat, Laurent Granjon

**Affiliations:** 1 CBGP, IRD, CIRAD, INRAE, Institut Agro, Université de Montpellier, Montpellier, France; 2 Centre de Recherche pour la lutte contre les Maladies Infectieuses Tropicales (CReMIT/TIDRC), Université d’Abomey-Calavi, Abomey-Calavi, Bénin; 3 IHPE, Univ Montpellier, CNRS, IFREMER, Univ Perpignan Via Domitia, Perpignan, France; 4 VITROME, Campus International IRD-UCAD de Hann, Dakar, Sénégal; 5 CBGP-BIOPASS 2, IRD, Campus IRD-ISRA de Bel-Air, Dakar, Sénégal; Swiss Tropical and Public Health Institute: Schweizerisches Tropen- und Public Health-Institut, SWITZERLAND

## Abstract

Schistosomiasis is a neglected tropical disease of public health significance. In view of its elimination as a public health problem by 2030, adopting a One Health approach is necessary, considering its multidimensional nature. Animal reservoirs, in particular, pose a significant threat to schistosomiasis control in Africa and beyond. In this study, we conducted a spatio-temporal survey of *Schistosoma* infections in small mammal communities and intermediate snail hosts in the vicinity of Lake Guiers in northern Senegal. Sampling campaigns were undertaken four times between April 2021 and August 2022 around eight villages. A total of 534 small mammals of four species, primarily Hubert’s multimammate mice *Mastomys huberti*, were captured. Out of 498 individuals examined, only 18 rodents (17 *M*. *huberti* and 1 *Arvicanthis niloticus*) were infected with schistosomes. The infection rates in *M*. *huberti* varied over time (prevalence range: 2.4% to 9.3%, intensity range: 4 to 132), and space (prevalence range: 3.1% to 40%, intensity range: 2 to 110) and were higher in adult hosts captured during or just after the rainy season, a time when older individuals dominate in rodent populations. Using a multi-locus molecular approach (*cox1* and ITS) on *Schistosoma* larvae (cercariae and miracidia) and adult worms, we identified *Schistosoma mansoni* as the most widespread species. We also detected *Schistosoma bovis* and *Schistosoma haematobium* in *M*. *huberti* from one locality (Temeye). Although no *Schistosoma* hybrids were found, the discovery of a male *S*. *mansoni* and a female *S*. *bovis* pair raises concerns about potential hybridization patterns that could occur in rodents. Finally, three snail species were found infected (25 *Biomphalaria pfeifferi*, 3 *Bulinus truncatus* and 1 *Bulinus senegalensis*) including with *S*. *mansoni*, *S*. *bovis*, *S*. *haematobium* and *S*. *haematobium x S*. *bovis* hybrids. Our findings highlight the spatial-temporal variations of *Schistosoma* infections in rodents and emphasize the need for fine-scale monitoring over time and space for effective One Health measures and ensuring the sustainability of schistosomiasis control efforts.

## Author summary

Schistosomiasis is a neglected tropical disease of major human and veterinary health concern. To ensure its sustainable control, the role of animal reservoirs that could maintain and spread the disease needs a better understanding. We investigated the spatio-temporal variations of *Schistosoma* infections in small mammal communities around Lake Guiers, northern Senegal. Small mammal species diversity was relatively low at transmission sites. Most infected rodents were found during or just after the rainy season, a time when older individuals dominate in rodent populations. From a spatial perspective, transmission was highly focalized and infection parameters (prevalence, intensity) highly variable, making it challenging to establish clear patterns. *Schistosoma mansoni*, responsible for human intestinal schistosomiasis, was the most widespread species found. However, sporadic infections with species of human (*S*. *haematobium*) and animal significance (*S*. *bovis*), including a case of mixed infections (*S*. *mansoni x S*. *bovis*) in rodents raises concerns about hybridization events that may occur in these hosts. Our findings confirm the role of rodents as reservoir or spill-over hosts for *Schistosoma* in northern Senegal and suggest that fine scale monitoring of animals at transmission sites could help to ensure the sustainability of schistosomiasis control.

## Introduction

Schistosomiasis is a neglected tropical disease (NTD) of major global health concern. According to the World Health Organization (WHO) over 250 million people are infected and approximately 200,000 deaths are reported annually [1,2]. The disease is endemic to Africa, Asia, the Middle East, South America and the West Indies, however the highest burden is recorded within sub-Saharan Africa [3]. The causative agents of the disease are digenean trematodes of the genus *Schistosoma*. In Africa, the major species of public health concern are *Schistosoma mansoni* and *Schistosoma haematobium*, causing intestinal and urogenital schistosomiasis, respectively [4]. Schistosomes have a complex life cycle involving specific freshwater snails as intermediate hosts (i.e., planorbs of the genus *Biomphalaria* for *S*. *mansoni* and *Bulinus* for *S*. *haematobium*) and a wide range of definitive vertebrate hosts, including human and other animals [5]. Infection occurs when the definitive host comes in contact with contaminated freshwater during daily activities. Larvae of the parasites, known as cercariae, released from their specific molluscs, actively penetrate the host skin and develop into adult worms. The pairing of male and female worms leads to the production of hundreds of eggs daily, which are excreted in the definitive host’s urine (*S*. *haematobium* infection) or faeces (*S*. *mansoni* infection), perpetuating the parasite’s life cycle. Some eggs remain trapped in the host’s tissues and are responsible for the pathology [4].

Despite substantial efforts to control schistosomiasis and the effectiveness of mass drug administration (MDA) using praziquantel (PZQ) in reducing global infections [6], the disease persists in endemic areas and has even re-emerged in previously controlled regions [7]. The establishment of schistosomiasis outside its endemic range, in Europe (Corsica, France), since 2013 [8,9] further highlights the need for comprehensive control strategies. In order to achieve the ambitious goals set by the WHO to eliminate schistosomiasis as a public health problem and ultimately complete interruption of transmission in selected regions by 2030 [3,10], a One Health approach is necessary [11,12]. This approach takes into account the multidimensional nature of this disease including the role of animal reservoirs and the potential multi-host, zoonotic transmission of schistosomes. Previous examples from the zoonotic species *Schistosoma japonicum* in Asia (infecting over 40 species) [13–16], but also *S*. *mansoni* in rodents in South America (Brazil) and the West Indies (Guadeloupe) [17–20] have illustrated the threat animal reservoirs and multi-host dynamics constitute to the sustainability of schistosomiasis control strategies. However, in Africa, our understanding of the host specificity of human- and animal-infective *Schistosoma* spp., the epidemiology of multi-host parasites and the role of animal reservoirs in human infection and disease control remains incomplete.

While *S*. *mansoni* is primarily associated to human infections, it has been found in over 50 different vertebrate hosts (see [21] for review) including a majority of non-human primates [22–25] and small mammals, particularly rodents [17–20,26–31]. Although their contribution to the transmission is poorly documented throughout Africa, small mammals have been found naturally infected with *S*. *mansoni* in several countries including Senegal [26–30], Ethiopia [32] and Kenya [31,33]. The main host species for *S*. *mansoni* are rodents of the Muridae family (*Mastomys huberti* and *Arvicantis niloticus* in Senegal, *Mastomys natalensis*, *Lophuromys flavopunctatus*, *Pelomys isseli* and *Aethomys kaiseri* in Kenya, whilst host species were not reported in the study from Ethiopia) and in rare cases shrews (*Crocidura olivieri* in Kenya).

Concerning *S*. *haematobium*, this species was until recently considered as a strictly human-specific parasite [34,35]. It has been found only on very rare occasions in other hosts [5,36,37], including rodents, with no evidence of a significant role in transmission. However, the epidemiological situation of urogenital schistosomiasis has become more complex in recent years, in particular due to the discovery of *Schistosoma* hybrids [38]. Indeed, humans have been diagnosed with urogenital schistosomiasis caused by hybrids between *S*. *haematobium* and *S*. *bovis* (a typical parasite of ruminants [26,27,31,39,40]) in several African countries [38,41–50], and following an outbreak in southern Europe [9,51,52], suggesting that host-specificity breakdown has already occurred, and further raising concerns on the zoonotic nature of African schistosomes. Moreover, the finding of a West African hybrid strain in Europe has alerted on the adaptive and invasive capabilities of these pathogens, likely to further complicate control strategies [9,51]. Recent studies showed that *S*. *haematobium* x *S*. *bovis* hybrids could also infect cattle in Benin [49] as well as rodents in Benin [53], Senegal [26], and in Corsica [54], thus supporting their zoonotic transmission on a local scale. However, compared to Benin where rodents and cattle seem to actively participate to transmission, only few rodents with low infection intensities and no cattle were found harbouring *Schistosoma* hybrids in Senegal [26] or Corsica [54]. This may suggest that the parasite multi-host transmission patterns may be dynamic and/or specific through space and potentially time [27].

To date, the global picture suggests that small mammals are suitable reservoirs for both intestinal and urogenital schistosomiasis caused by hybrids. Rodents are of particular interest for several reasons. First, schistosomes have a strong affinity for rodents, which are commonly used in laboratory settings to maintain various *Schistosoma* species [55]. Second, their capacity to live as commensals in close contact with humans make them good candidates as main reservoirs of zoonotic diseases [56,57]. Third, their large population sizes, wide distributions and high reproductive capacities, further contribute to their potential as key hosts in the transmission of zoonotic pathogens [12,58]. Last, rodents are unique in that they are the only known hosts to carry a diversity of *Schistosoma* spp. that infect both humans (*S*. *mansoni*, *S*. *haematobium* and *S*. *haematobium* x *S*. *bovis* hybrids) and animals (*S*. *bovis* and *S*. *haematobium* x *S*. *bovis* hybrids, *Schistosoma rodhaini*). Rodents, claimed as “biotic hubs" [26,31], may provide a favourable environment for genetic interactions and/or hybridization of *Schistosoma* species, resulting in the emergence of new strains or hybrids with varying morbidity, host range and drug susceptibility [27,38,59]. All together this highlights the importance of understanding the role of rodents in the global burden of schistosomiasis for effective control strategies.

In this study we conducted a spatio-temporal survey of small mammal communities on the shores of Lake Guiers in Senegal, a endemic region for human [45,47,60,61] and animal [26,27,29,45] schistosomes. Our aim was to describe potential variations in infection levels with *Schistosoma* species across space and time and establish links with host species and individual characteristics, which are important parameters to help adapting the management of the disease.

## Material and methods

### Ethical statement

The “Centre de Biologie pour la Gestion des Populations” (CBGP) carries out research activities on rodent-parasite interactions and their consequences for health. The establishment holds an approval on using animals for scientific purposes (F34-169-001, prefectural decree n°24 XIX-042). The project obtained a favourable advice of the Comité Consultatif Éthique pour la Recherche en Partenariat (CCERP) of the Institut de Recherche pour le Développement (IRD) (dated 29 November 2021). Trapping campaigns were organized after informing the village chiefs of the area about the objectives of the project, and obtaining explicit consent from local authorities and land owners. All targeted small mammals are classified as "Least Concern" (LC) by the International Union for Conservation of Nature (IUCN) Red List. The examined animals were treated in accordance with published guidelines on animal welfare and the use of wildlife in research [62]. Captures of non-target species were rare and specimens caught unintentionally were released at their point of capture. In the absence of a national legislation of Senegal for the implementation of the Nagoya Protocol on Access and Benefit-Sharing (ABS), we obtained an exemption from authorization of access and use of genetic resources (n° 001105) issued by the Competent National Authority (the National Focal point on ABS).

### Hosts sampling through space and time

**Fig 1:**
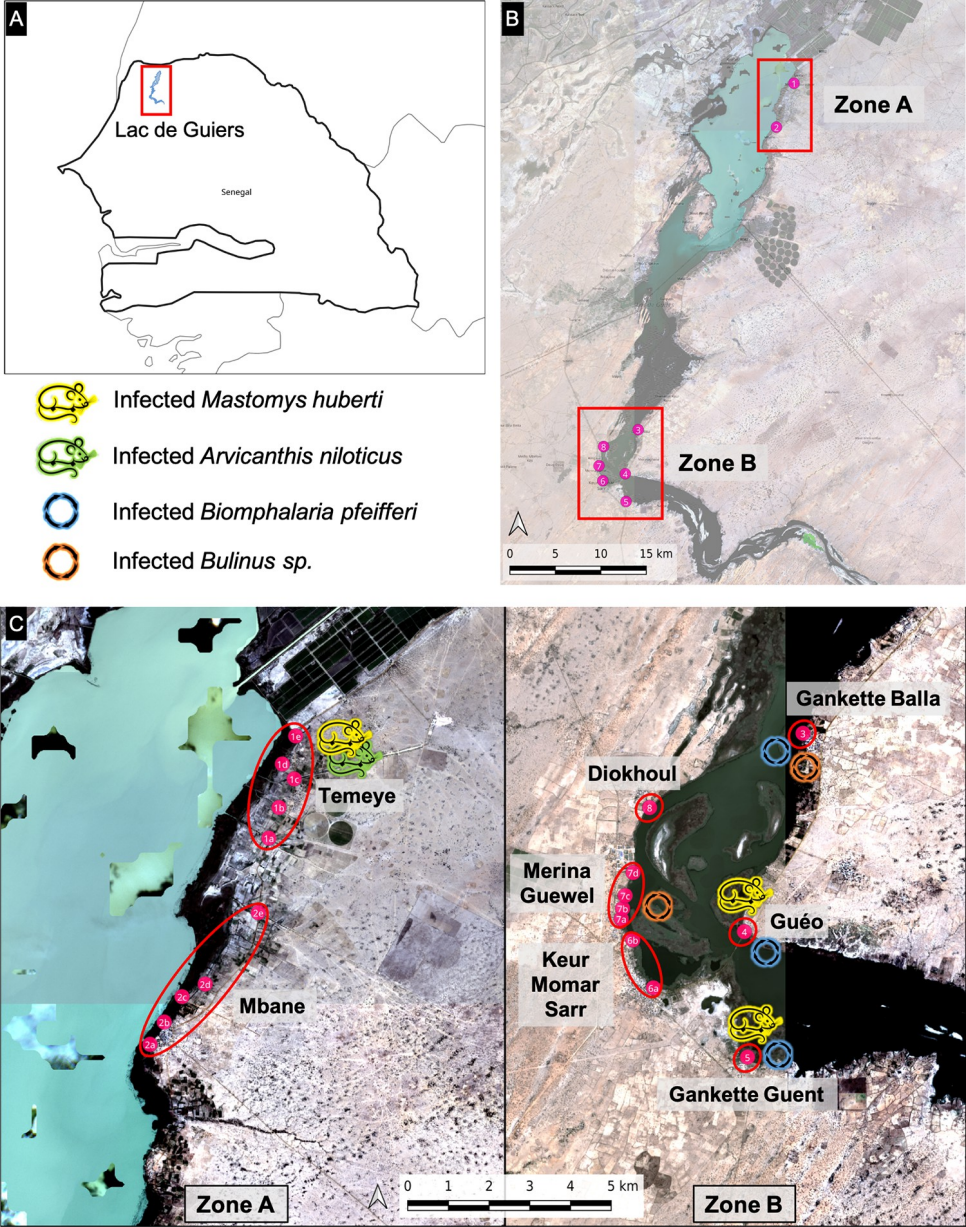
Map of the study area showing locations sampled during the survey. **A)** Geographical situation of the Lake Guiers in northwestern Senegal. **B)** Localization of zones A and B and main villages sampled around the lake: 1. Temeye, 2. Mbane, 3. Gankette Balla, 4. Guéo, 5. Gankette Guent, 6. Keur Momar Sarr, 7. Merina Guewel, and 8. Diokhoul. **C)** Sampling sites within or adjacent to each village including five sites for Temeye (1a: Canal Sall, 1b: Lewa, 1c: Lewa-Soubalo, 1d: Soubalo, 1e: North), five sites for Mbane (2a: South, 2b: Center, 2c: North_A, 2d: North_B, 2e: North_C), a unique site for Gankette Balla (3), Guéo (4) and Gankette Guent (5), two sites for Keur Momar Sarr (6a: KMS_South, 6b: Feto), four sites for Merina Guewel (7a: Merina Guewel_Center 7b: Merina Guewel 7c: Takh, 7d: Ringaye) and a unique site for Diokhoul (8). The figure was produced using images from Sentinel-2 (January 2022, theia-land.fr) and Natural Earth (naturalearthdata.com) under Qgis (http://www.qgis.org). The base layer of the map (i.e., the country border shape) was downloaded from https://www.naturalearthdata.com/downloads/50m-cultural-vectors/. It contains information from OpenStreetMap and OpenStreetMap Foundation, which is made available under the Open Database License.

Fieldwork was carried out in the vicinity of Lake Guiers in northwestern Senegal (Fig 1A). The climate is of Sahelian type, characterized by an 8–9 month-long dry season and a short rainy season between July and October. Fieldwork was conducted over two years at four occasions, namely in April and November 2021, and in February-March and August 2022, the latter corresponding to the rainy season. For the purpose of this study and because of differences in land usage and access to freshwater sources, two geographical zones separated by roughly 50km were selected. Zone A located north-east of the lake (Fig 1B) is mainly characterized by irrigated crop fields and a large network of irrigation canals serving as potential schistosomiasis transmission sites. Zone B located south of the lake (Fig 1B) relies on traditional cultivation and livestock breeding with schistosomiasis transmission sites suspected to be on the shores of the lake itself. These two zones were also selected because of previous data indicating the presence of *Schistosoma* spp. and/or their hybrids in rodent populations [26,27]. Two main villages were targeted in zone A (1: Temeye and 2: Mbane), and six villages in zone B (3: Gankette Balla, 4: Guéo, 5: Gankette Guent, 6: Keur Momar Sarr, 7: Merina Guewel, and 8: Diokhoul) (Fig 1B). Within or adjacent to these villages, sampling sites corresponding to potential transmission foci were defined (based on knowledge of the frequentation of these sites by the local populations) where small mammals were captured (Fig 1C). The exact position with GPS coordinates of each trapping site is available in S1 File.

### Trapping of small mammals

Small mammals were captured using locally made wire-mesh live traps (8.5 x 8.5 x 26.5 cm). Trapping sites correspond to various access points to water (including holes in the riparian vegetation composed of *Typha* sp. and *Phragmites* sp.) serving for laundry, bathing and fishing activities as well as irrigation canals and livestock watering sites. The traps were baited with local peanut butter and placed at the end of the afternoon at 5–10 meter intervals for 1 to 3 nights per site and per sampling period. The traps were systematically inspected and removed the following morning to avoid the capture of non-target species and any untimely removal in these frequented sites. Live small mammals were returned to the laboratory, kept in a quiet, shaded environment until they were euthanized for necropsy (within four hours to minimize the time they spend captive). Information on the date, location, habitat and daily results obtained at each trapping site were stored in the “Small Mammals” database (https://doi.org/10.15454/WWNUPO) hosted by the CBGP.

### Post-mortem examinations

Small mammals were euthanized by intraperitoneal injection of sodium thiopental (300 mg/kg body weight) and death was confirmed after cervical dislocation and the absence of pedal reflex. Individuals were identified to the species level based on morphological, ecological and biogeographical knowledge [63]. For each specimen we recorded body weight, gender, reproductive status and external measurements (head and body, tail, hindfoot, and ear lengths) according to standard procedures [63]. Age class (adult/juvenile) was inferred from weight distributions, body length and reproductive status [63]. To account for potential biases in the capture rate (CR) through space and time, a “disturbance index” corresponding to the ratio between half the number of “inactive” traps (i.e., traps closed without any capture, traps found missing, or traps occupied by individuals of non-target species) and the number of trap nights was calculated [64]. Because this index showed low variation across space and over time (see results) the CR was used as a proxy of the relative abundance of each species. It was calculated per night for each trapping site as the number of animals captured divided by the total number of set traps (i.e., the trapping effort) [63].

### Diagnosis of *Schistosoma* infections in small mammals

The presence and abundance of adult worms were used to determine the prevalence and intensity of *Schistosoma* spp. infection in small mammals, respectively. We used the hepatic perfusion technique [65] (slightly adapted to fieldwork conditions) and dissections to diagnose and extract the worms from small mammals blood circulating system. Briefly, a 50 ml syringe was used to perfuse the euthanized animals with a physiological solution of sodium citrate (7.5‰) and sodium chloride (8.5‰). The perfusate injected in the left ventricle was collected from the hepatic portal vein and filtered through a polyamide cloth. If any, *Schistosoma* adult worms were counted and pairs separated under a binocular magnifying glass. Specific organs of each host examined at necropsy (liver, mesenteries and large intestine) were carefully inspected to identify any remaining worms even if the hepatic perfusion was negative. Schistosomes were individually stored at room temperature in annotated 2 ml Eppendorf tubes containing 95% ethanol during fieldwork, then at 4°C upon arrival at the laboratory. Parasite eggs collected from the granulomatous livers of hosts with signs of infection (presence of worms, granulomatous formations due to eggs) were tested for miracidia hatching. Briefly, the liver and the large intestine where *Schistosoma* eggs accumulate were grounded through a colander and abundantly washed with NaCl solution (8.5‰). The suspension obtained was filtered through a series of metal sieves of decreasing mesh size (425, 180, 106 and 45 μm) to concentrate potential eggs. The final sieve (45 μm) was rinsed over a Petri dish with bottled spring water at room temperature to recover the eggs. Miracidia hatching from eggs was stimulated in freshwater under indirect sunlight or a lamp [66]. After 20 minutes and up to 6h, the Petri dishes were inspected for the presence of miracidia under a binocular magnifying glass. Larvae were individually collected in 2μL using a micropipette before being rinsed in a drop of bottled spring water and transferred onto a Whatman Indicating FTA Classic card (GE Healthcare Life Sciences) for DNA storage [67,68].

### Intermediate hosts sampling and parasitological diagnosis

Schistosome intermediate host snails were collected following an opportunistic, non-standardized sampling procedure within or adjacent to some of the small mammal capture sites. We favoured open freshwater sources with abundant vegetation mostly composed of *Ceratophyllum* sp. as favourable habitats for the snails [69]. Snails were collected using a scoop net and species were identified using shell morphology [70]. Only potential hosts for schistosomes were kept (*Bulinus* spp. and *Biomphalaria pfeifferi*). Infection status and associated prevalence in snails were determined after inducing the emission of *Schistosoma* cercariae. Each snail was abundantly washed with freshwater to avoid parasite cross-contaminations and individually transferred to a tube containing bottled spring water. Snails where exposed to indirect sunlight or artificial light over the day to take into account differences in chronobiological rhythms of different *Schistosoma* species so that patent snails release cercariae [67]. The cercariae from positive snails were individually pipetted in 2μL under a binocular magnifying glass onto Whatman Indicating FTA Classic cards for DNA storage [67,68]. Positive molluscs were stored individually while remaining, non-patent molluscs were pooled and stored in 95% ethanol.

### Molecular characterisation of the parasites

DNA from adult worms kept in ethanol was extracted using the DNeasy Blood and Tissue Kit (QIAGEN) following the manufacturer’s instructions. DNA from larvae (miracidia from rodents and cercariae from snails) preserved on Whatman Indicating FTA Classic cards was processed as described previously [71]. The samples (miracidia, cercariae and adult worms) were first characterized by a rapid diagnostic multiplex polymerase chain reaction (RD-PCR) targeting a partial fragment of the mitochondrial cytochrome c oxidase subunit 1 (*cox1*) [72,73]. Briefly, the universal forward primer Asmit1 (5′-TTTTTTGGTCATCCTGAGGTGTAT-3′) and three species-specific reverse primers, namely ShR (5′-TGATAATCAATGACCCTGCAATAA-3′) for *S*. *haematobium* (amplicon size: 543 bp); SbR (5′-CACAGGATCAGACAAACGAGTACC-3′) for S. *bovis* (amplicon size: 306 bp) and SmR (5′-TGCAGATAAAGCCACCCCTGTG-3′) for *S*. *mansoni* (amplicon size: 375 bp) were used in this study. Reactions were performed in 12.5μL containing 2 μL of DNA template, 1× of Dream Taq green buffer (including 2 mM of MgCl_2_), 0.2 mM of dNTP, 0.4 μM of each primer, 1 unit of Dream Taq (Thermo Fisher Scientific, France) and ultra-pure water. Cycling parameters for *cox1* RD-PCR consisted of an initial denaturation at 95°C for 5 min, followed by 40 cycles at 95°C for 30 s, 58°C for 30 s, and 72°C for 1 min, with a final 7 min extension at 72°C. Because of potential hybridization between *Schistosoma* species, DNA was also amplified and sequenced for the complete (981 bp) nuclear ribosomal DNA internal transcribed spacer (ITS). We used the forward ETTS1 (5′-TGCTTAAGTTCAGCGGGT-3′) and the reverse ETTS2 primers (5′-AACAAGGTTTCCGTAGGTGAA-3′) [74] and the same PCR reactions as described above. PCR cycling parameters for the ITS gene differed from the *cox1* PCR only by increasing the elongation step to 1 min 30 sec. Four microliter of each PCR products were run on a 1.5% agarose gel for 45 min at 120V to confirm successful amplification and verify amplicon size used to discriminate each species (*cox1*). The ITS PCR products and a subset of randomly chosen *cox1* amplicons (to confirm RD-PCR profiles) were sent to Eurofins MWG (Germany) for purification and sequencing using original PCR primers.

### DNA (*cox1* and ITS) sequencing analysis

Successful sequences were assembled and manually edited using Sequencher v.4.5 (http://genecodes.com) to remove ambiguities and sequencing errors. The cleaned sequences were aligned with MUSCLE on MEGA version 7.0.26 [75,76]. Species assignment was performed by comparison with sequences available in GenBank. The identity of the sequence (species and gene) was confirmed using the basic local alignment search tool (BLAST). As hybrids of first generation generally exhibit both parental nuclear rDNA ITS copies, appearing as double peaks on chromatograms, known polymorphic positions between species observed on the ITS gene were verified by visualisation of the original sequence chromatograms to identify heterozygosity [77]. Therefore, hybrids are detected either when the ITS marker shows heterozygous pattern at specific mutation points and/or if ITS and *cox1* markers are discordant in species assignation. The *cox1* and ITS sequences from individual samples are available in S3 File and were deposited in GenBank (NCBI) under accession numbers PP658465—PP658508 (*cox1*) and PP658638—PP658972 (ITS).

### Statistical analyses

Generalized linear mixed models (GLMMs) were carried out to evaluate spatio-temporal variations in rodent populations. GLMMs are particularly useful because they allowed us to account for both fixed effects, such as the influence of time and location, and random effects, such as variability between sampling periods or villages. This approach provides a more flexible and robust framework for analysing complex ecological data with repeated measures and hierarchical structure. Three separate models were applied to trap-level data, incorporating both temporal (i.e., ‘sampling period’) and spatial (i.e., ‘zone’, ‘village’) predictors. The models aimed to analyze, as dependent variables: (i) the rodent capture rate (i.e., whether at least one rodent was captured or not; model #1), (ii) the rodent sex (i.e., whether the individual is a female or a male; model #2), and (iii) the rodent age class (i.e., whether the individual is a juvenile or an adult; model #3). Using a three-model approach is essential because each dependent variable—capture presence, sex, and age class—represents a distinct biological aspect of the population and requires separate statistical treatment. This allowed us to address different ecological questions, tailored to the unique structure of each response variable. For each model, we considered a binomial distribution and a model selection approach was performed, using the Akaike information criterion with correction for samples of finite size (AICc) as a goodness-of-fit indicator [78–80]. All predictors were included in the starting models, and the “sampling site” was added as a random factor. Models with all possible combinations of the terms included in the starting model were generated and those with a ΔAICc < 2 with respect to the model with the lowest AICc were selected. Among them, the most parsimonious was chosen based on the fewest number of predictors kept. Deletion testing and log-likelihood ratio (LRT) tests determined the significance of explanatory variables. The assumptions of each final model were checked graphically, by an analysis of their residuals. When needed, post-hoc comparisons were carried out with pairwise Wilcoxon rank sum tests (PWT, 95% family-wise confidence level). We carried out the whole procedure at both interspecific and intraspecific levels (for dominant species, see below) for model #1, but only at intraspecific level for model #2 and model #3. The summary of the most parsimonious GLMMs finally selected is presented in S1 Table. *Schistosoma* prevalence (i.e., percentage of infected hosts within the sampled host population) were compared over time (sampling periods), across space (zones, villages), and following host-related factors (‘age class’ and ‘gender’) using a Pearson’s χ^2^ test or a Fisher Exact test when sample size expected frequencies was less than 5. The intensity of infection (i.e., number of worms per infected host) in rodents were compared according to the same variables using *(i)* either a Kruskal-Wallis (KW) test for variables with more than two modalities (sampling periods, villages), followed by PWT in case KW tests were significant, or *(ii)* a Wilcoxon—Mann Whitney test for variables with only two modalities (zone, gender). Statistical analyses were performed using DHARMa [81], MuMIn v1.43.6 [82], and lme4 v1.1–8 [83] R packages under R version 4.4.0 (http://www.rproject.org) and BiostaTGV (http://biostatgv.sentiweb.fr). For all tests, statistical differences were considered significant when p < 0.05.

## Results

### Small mammal trapping through time and space

A total of 534 small mammals were captured over the course of 4122 trap nights, representing an overall CR of 13.0% over two years. Noticeably, 710 of the 4122 overall trap nights (i.e., >17%) were considered as inactive as they were found either closed without any capture (n = 530), missing (n = 42), or occupied by individuals of non-target species (n = 138). The majority of by-catch individuals were unidentified anuran amphibians (n = 83), followed by birds (*Amaurornis flavirostra* and *Gallinula chloropus*, n = 48) and fishes (catfish and tilapia, n = 7) that were immediately released (S1 File). Our disturbance index showed low variation over sampling periods (range: 7.8% in April 2021 to 10.2% in November 2021) and across villages (range: 7.5% in Mbane to 10.8% in Keur Momar Sarr) thus no correction was applied to the CR calculation based on the total trapping effort (S1 File).

Overall, the targeted rodent species caught comprised 356 *M*. *huberti*, 137 *A*. *niloticus*, and 2 *Taterillus pygargus*. Additionally, 39 *Crocidura viaria* shrews were trapped. Species-related CR was in favour of *M*. *huberti*, which was clearly dominant along the partially flooded shores of the lake and along irrigation canals, often overgrown with reeds, followed by *A*. *niloticus*, which was well represented in crop fields and gardens adjacent to water bodies, then *C*. *viaria* and *T*. *pygargus* (Table 1). Considering all species, model #1 revealed that the overall CR significantly varied over sampling periods from April 2021 to August 2022 (LRT = 28.01, df = 3, p = 3.6e-06). This change in CR over time was significant for *M*. *huberti* (LRT = 16.53, df = 3, p = 0.0009) in February-March and August 2022 compared to both April and November 2021. The CR significantly varied for *A*. *niloticus* (LRT = 18.24, df = 3, p = 0.01) only between April 2021 and the other sampling periods (PWT, p < 0.05). Noticeably, after April 2021, crop fields and gardens adjacent to water bodies were less intensively sampled because of the low probability of finding infected individuals there: this may explain the lower CR for *A*. *niloticus* observed after the first sampling period.

**Table 1:** Numbers of trap nights and individuals captured for each species at each sampling period, with the capture rate in parentheses.

Sampling periods	April 2021	November 2021	February-March 2022	August 2022	Total
**Nb. trap nights**	835	1107	1222	958	4122
**Nb. closed-empty**	109	167	150	104	530
**Nb. missing traps**	8	9	10	15	42
**By-catch**	14	49	28	47	138
**Disturbance index**	7.8%	10.2%	7.7%	8.7%	8.6%
***Mastomys huberti***	91 (10.9%)	121 (10.9%)	90 (7.4%)	54 (5.6%)	356 (8.7%)
***Arvicanthis niloticus***	60 (7.2%)	22 (2.0%)	37 (3.0%)	18 (1.9%)	137 (3.3%)
***Crocidura viaria***	8 (1.0%)	17 (1.5%)	10 (0.8%)	4 (0.4%)	39 (0.9%)
***Taterillus pygargus***	0	1 (0.1%)	1 (0.1%)	0	2 (0.5%)
**Total captures**	159 (19.0%)	161 (14.5%)	138 (11.3%)	76 (7.9%)	534 (13.0%)

At the spatial level, we did not find significant differences in the overall CR between the geographic zones. However, model #1 suggested significant variations of overall CR between villages within and across geographic zones (LRT = 18.24, df = 7, p = 0.011) with overall values ranging from 5.5% in Guéo to 22.3% in Mbane (Table 2). The number of species captured varied according to the geographical zones. Four species were captured in zone A and only *M*. *huberti* and *A*. *niloticus* trapped in zone B (Table 2). Variations of CR (model#1) between villages were significant at the intraspecific level for *M*. *huberti* (LRT = 25.38, df = 7, p = 0.0007) and *A*. *niloticus* (LRT = 20.88, df = 7, p = 0.004). Nonetheless, variations of CR seemed to suggest opposite trends between *M*. *huberti* and *A*. *niloticus*, the former being overall more abundant in zone B, while the latter being more abundant in zone A. It is however worthy to note that the highest capture rate for *A*. *niloticus* was found in only one locality of zone A where several sites adjacent to crops fields and gardens where sampled (Mbane), *M*. *huberti* being the dominant species in all other localities (Table 2 and S1 File).

**Table 2:** Summary of the number of individuals captured through space. Capture rates are indicated in parentheses. The code in parentheses for each site refers to Fig 1.

Zone-Village	Trap nights	*Mastomys huberti*	*Arvicanthis niloticus*	*Crocidura viaria*	*Taterillus pygargus*	Total
A-Temeye (1)	973	48 (4.9%)	19 (2.0%)	16 (1.6%)	2 (0.2%)	85 (8.7%)
A-Mbane (2)	640	31 (4.8%)	89 (13.9%)	23 (3.6%)	0	143 (22.3%)
B-GKB (3)	200	12 (6.0%)	10 (5.0%)	0	0	22 (11.0%)
B-Guéo (4)	709	39 (5.5%)	0	0	0	39 (5.5%)
B-GKG (5)	592	75 (12.7%)	2 (0.3%)	0	0	77 (13.0%)
B-KMS (6)	355	67 (18.9%)	3 (0.8%)	0	0	70 (19.7%)
B-MGW (7)	503	74 (14.7%)	12 (2.4%)	0	0	86 (17.1%)
B-Diokhoul (8)	150	10 (10.5%)	2 (2.1%)	0	0	12 (8.0%)

### Small mammal age and sex population structure

As both *C*. *varia* and *T*. *pygargus* were represented by low numbers of individuals subsequent analyses of population sex- (model#2) and age- (model#3) structure focused only on *M*. *huberti* and *A*. *niloticus*. We unambiguously attributed 355 and 339 *M*. *huberti*, and 137 and 128 *A*. *niloticus* to an age and sex class, respectively. The deficit in the number of individuals attributed to a particular sex in comparison to those attributed to an age class arises from the capture and immediate release of very young individuals which did not have time to become infected with schistosomes. Although their sex was not recorded, these individuals were classified as juveniles. The proportion of juveniles within each species significantly varied over time (*M*. *huberti*: LRT = 13.03, df = 3, p = 0.005; *A*. *niloticus*: LRT = 18.55, df = 3, p = 0.0003) with an overall lower proportion of juveniles in August 2022 compared to both April 2021 and February-March 2022 for *M*. *huberti* (PWT, p-values < 0.05) and in April and November 2021 compared to both February-March or August 2022 for *A*. *niloticus* (PWT, p-values < 0.05).

At the spatial scale, no significant difference was observed between the overall proportion of juveniles between zone A and B, either for *M*. *huberti* or for *A*. *niloticus*, given this variable was not kept in the most parsimonious model selected (Table 3). Furthermore, our GLMMs did not reveal any significant variation of the sex ratio between sampling periods, villages or geographic zones, neither in *M*. *huberti* nor in *A*. *niloticus*. The summary of the GLMMs information is presented in S1 Table.

**Table 3:** Population age-structure of *M*. *huberti* and *A*. *niloticus* populations over time and across space. The proportion of juveniles is indicated in parentheses.

Zone	Species	Number of juveniles / total number of individuals captured				
		April 2021	November 2021	February-March 2022	August 2022	Total
A	*Mastomys huberti*	2/17 (11.8%)	2/19 (10.5%)	6/24 (25.0%)	0/18 (0%)	10/78 (12.8%)
	*Arvicanthis niloticus*	5 /50 (10.0%)	0/15 (0%)	11/28 (39.3%)	7/15 (46.7%)	23/108 (21.3%)
B	*Mastomys huberti*	17/74 (23.0%)	16/102 (15.7%)	14/65 (21.5%)	2/36 (5.6%)	49/277 (17.7%)
	*Arvicanthis niloticus*	0/10 (0%)	1/7 (14.3%)	2/9 (22.2%)	0/3 (0%)	3/29 (10.3%)
A & B	*Mastomys huberti*	19/91 (20.9%)	18/121 (14.9%)	20/89 (22.5%)	2/54 (3.7%)	59/355 (16.6%)
	*Arvicanthis niloticus*	5/60 (8.3%)	1/22 (4.5%)	13/37 (35.1%)	7/18 (38.9%)	26/137 (19.0%)

### *Schistosoma* spp. infection in small mammal communities

Amongst the 534 specimens trapped, 498 were examined for *Schistosoma* infection including 336 *M*. *huberti*, 126 *A*. *niloticus*, 35 *C*. *viaria* and 1 *T*. *pygargus*. In total, *Schistosoma* trematodes were only detected in 18 rodents, representing an overall prevalence of 3.6% (Table 4 and S2 File). No *Crocidura* shrews or *Taterillus* gerbils examined were found infected with schistosomes, and a single *A*. *niloticus* individual (adult female) was found to be infected (prevalence: 0.8%). Infected *M*. *huberti* (prevalence: 5.1%) were exclusively adult individuals caught close to freshwater sources (<5 meters). There was no significant difference of prevalence between females (10/154) and males (7/182) *M*. *huberti* (χ^2^ = 1.21, df = 1, p = 0.27*)*. Overall, a total of 191 adult worms were recovered from rodents across all hosts, sampling sites and sampling periods (median: 4, range: 1–52 among carrier hosts): 186 in *M*. *huberti* (median: 2, range: 1–52) and 5 in *A*. *niloticus* (Table 4). The intensity of infection in *M*. *huberti* was not significantly different between male (median: 5, range: 1–52) and female (median: 2, range: 1–39) rodents (U = 25.5; p = 0.37). Six *M*. *huberti* individuals (35.3%) harboured single male worms, whereas 11 (64.7%) presented at least one couple of paired male and female schistosomes. The overall parasite’s sex ratio was in favour of males (102/89 = 1.15), including at the host species level in *M*. *huberti* (99/87 = 1.14) and *A*. *niloticus* (3/2 = 1.50). In total, 11 hosts (10 *M*. *huberti* and 1 *A*. *niloticus*) presented eggs in the liver and the mesenteries from which we hatched and collected live miracidia. Interestingly, one host (ID: JKIN_050) with a unique adult male *Schistosoma* showed signs of infection (granuloma to the liver). The successful hatching of miracidia proved the presence of an unseen female worm or an ancient infection. In two other hosts, which had worm pairs, no eggs or miracidia were found, indicating recent infections with sexually immature female worms. Details at the individual host level are available in S2 File.

**Table 4:** Prevalence and intensity of *Schistosoma* infection in rodents over time and across space. Only sites where infected hosts were detected are included in this table (the code in parentheses for each site refers to Fig 1). *M*. *huberti*: *Mastomys huberti*, *A*. *niloticus*: *Arvicanthis niloticus*, P: prevalence; I: intensity (median; range). “N-I” refers to the presence of only non-infected individuals (S2 File).

Zone–Village-Site	April 2021	November 2021	February-March 2022	August 2022	Overall periods
A-Temeye-Canal Sall (1a)	No data	*M*. *huberti* P: 1/3; I: 23	N-I	*M*. *huberti* P: 2/7; I: 27 (14; 1–26)	*M*. *huberti* P: 3/11; I: 50 (23; 1–26)
A-Temeye-Lewa (1b)	N-I	*M*. *huberti* P: 1/1; I: 1	N-I	*M*. *huberti* P: 1/1; I: 1	*M*. *huberti* P: 2/5; I: 2 (1)
A-Temeye-Lewa-Soubalo (1c)	No data	*M*. *huberti* P: 1/1; I: 5	N-I	*M*. *huberti* P: 1/7; I: 1	*M*. *huberti* P: 2/12; I: 6 (3; 1–5)
A-Temeye-Soubalo (1d)	N-I	*A*. *niloticus* P: 1/3; I: 5	*M*. *huberti* P: 2/6; I: 4 (2)	N-I	*M*. *huberti* P: 2/16; I: 4 (2)
					*A*. *niloticus* P: 1/12; I: 5
B-Guéo (4)	*M*. *huberti* P: 2/12; I: 7 (4; 1–6)	*M*. *huberti* P: 4/15; I: 103 (25; 2–52)	N-I	N-I	*M*. *huberti* P: 6/38; I: 110 (8; 1–52)
B-Gankette Guent (5)	*M*. *huberti* P: 1/25; I: 1	N-I	N-I	*M*. *huberti* P: 1/3; I: 13	*M*. *huberti* P: 2/64; I: 14 (7; 1–13)
Overall zone A	N-I	*M*. *huberti* P: 3/19; I: 29 (5; 1–23)	*M*. *huberti* P: 2/22; I: 4 (2)	*M*. *huberti* P: 4/18; I: 29 (1; 1–26)	*M*. *huberti* P: 9/76; I: 62 (2; 1–26)
		*A*. *niloticus* P: 1/15; I: 5			*A*. *niloticus* P: 1/99; I: 5
Overall zone B	*M*. *huberti* P: 3/74; I: 8 (1; 1–6)	*M*. *huberti* P: 4/88; I: 103 (25; 2–52)	N-I	*M*. *huberti* P: 1/36; I: 13	*M*. *huberti* P: 8/260; I: 124 (8; 1–52)
Overall zone A & B	*M*. *huberti* P: 3/91; I: 8 (1; 1–6)	*M*. *huberti* P: 7/107; I: 132 (10; 1–52)	*M*. *huberti* P: 2/84; I: 4 (2)	*M*. *huberti* P: 5/54; I: 42 (1; 1–26)	*M*. *huberti* P: 17/336; I: 186 (2; 1–52)
		*A*. *niloticus* P: 1/22; I: 5			*A*. *niloticus* P: 1/126; I: 5

Schistosomes were found in rodents during all sampling periods with variations in the overall prevalence and intensity (Table 4). The overall prevalence in *M*. *huberti* ranged from 2.4% in February-March 2022 to 9.3% in August 2022. In August 2022 and November 2021, when the majority of infected rodents were found, the intensity of infection could be substantial, reaching 23 and up to 52 worms in some individuals, a pattern not observed in other occasions (Table 4). Interestingly, August was also the time when rodents’ capture rates and the proportion of juveniles in *M*. *huberti* were the lowest. However, probably due to a limited number of infected hosts and the high variance between hosts and over time, we did not find any significant difference in the overall prevalence (Fisher exact test: p = 0.24) and intensity (KW = 3.34, p = 0.34) across sampling periods in *M*. *huberti* (Table 4).

At the spatial scale, schistosome distribution in rodents was highly focalised. Infections occurred in both geographic zones but only within particular localities (3 villages out of the 8 studied) and within particular sites (6 sites out of 20) sometimes separated from the nearest, “non-infected” one, by only a few hundreds of meters. Zone A harboured more sites with infected rodents (n = 4) than zone B (n = 2), and the overall prevalence in *M*. *huberti* was significantly higher there also (Fisher exact test: p = 0.005). In contrast, differences of infection intensity between zones were not significant (U = 24.0, p = 0.26). At the scale of the villages, the prevalence in *M*. *huberti* significantly varied between 3.1% in Gankette Guent, 15.8% in Guéo, and 20.5% in Temeye (Fisher exact test: p = 0.01) and reached up to 40% in the specific site of Temeye Lewa (Table 4). The intensity of infection was highly variable between hosts but did not differ significantly across space in *M*. *huberti* (village: KW = 1.96; p = 0.38), probably due to a relatively low sample size.

### Snail intermediate hosts sampling and *Schistosoma* infection

A total of 599 intermediate host snails were collected throughout our study (Table 5). Overall, we identified 318 *Biomphalaria* sp. (*Bi*. *pfeifferi)*, and 281 *Bulinus* sp. (159 *Bu*. *truncatus*, 62 *Bu*. *globosus*, 43 *Bu*. *senegalensis* and 17 *Bu*. *forskalii*) according to shell morphology. The abundance, diversity and infectivity of snails were not compared over time and across space because of our non-standardized sampling. However, amongst all, 29 snails (4.8%) shed schistosome cercariae (Table 5). The majority of infection occurred in *Bi*. *pfeifferi* followed by *Bu*. *senegalensis* and *Bu*. *truncatus* with an overall prevalence of 7.9% (25/318), 2.3% (1/43) and 1.9% (3/159), respectively (Fisher exact test: p = 0.01). *Biomphalaria pfeifferi* were found infected at all sampling periods except in November 2021. Infected *Bulinus* spp. were found in February-March and August 2022 (*Bu*. *truncatus* during both sampling periods, *Bu*. *senegalensis* in August only).

**Table 5:** *Schistosoma* prevalence in intermediate host snail species sampled through time and space. The codes in parentheses for each site refers to Fig 1). Positive snails (shedding schistosome cercariae) are indicated with the symbol +. Other sites, not mentioned in this table, were not sampled for snails. Bi.: *Biomphalaria*, *Bu*.: *Bulinus*.

Zone-village-site	April 2021	November 2021	February-March 2022	August 2022
**A-Teme-Canal sall (1a)**	Not sampled	*Bu*. *truncatus* (0/1) *Bu*. *globosus* (0/16) *Bu*. *senegalensis* (0/6) *Bu*. *forskalii* (0/11)	Not sampled	Not sampled
**A-Temeye-Lewa-Soubalo (1c)**	Not sampled	*Bu*. *globosus* (0/1) *Bu*. *forskalii* (0/2)	Not sampled	Not sampled
**A-Temeye-Soubalo (1d)**	*Bu*. *truncatus* (0/21)	*Bi*. *pfeifferi* (0/21) *Bu*. *truncatus* (0/1)	Not sampled	Not sampled
**A-Temeye-North (1e)**	*Bi*. *pfeifferi* (0/27) *Bu*. *globosus* (0/5) *Bu*. *forskalii* (0/1)	*Bu*. *globosus* (0/9) *Bu*. *senegalensis* (0/5)	Not sampled	*Bi*. *pfeifferi* (0/10) *Bu*. *truncatus* (0/1) *Bu*. *globosus* (0/25) *Bu*. *senegalensis* (0/30) *Bu*. *forskalii* (0/2)
**A-Mbane Center (2b)**	Not sampled	*Bi*. *pfeifferi* (0/20) *Bu*. *forskalii* (0/1)	Not sampled	Not sampled
**B-Gankette Balla (3)**	Not sampled	Not sampled	*Bi*. *pfeifferi* (0/17) ***+*** *Bu*. *truncatus* (2/14) *Bu*. *globosus* (0/1)	***+*** *Bi*. *pfeifferi* (1/11) *Bu*. *truncatus* (0/2) *Bu*. *senegalensis* (0/1)
**B-Guéo (4)**	***+*** *Bi*. *pfeifferi* (7/37) *Bu*. *truncatus* (0/100)	Not sampled	***+*** *Bi*. *pfeifferi* (10/91) *Bu*. *truncatus* (0/16) *Bu*. *globosus* (0/3)	***+*** *Bi*. *pfeifferi* (1/6) *Bu*. *truncatus* (0/1)
**B-Gankette Guent (5)**	*Bi*. *pfeifferi* (0/24)	*Bi*. *pfeifferi* (0/7)	*Bu*. *truncatus* (0/1) *Bu*. *globosus* (0/1)	***+*** *Bi*. *pfeifferi* (6/47)
**B-Merina Guewel-Takh (7c)**	Not sampled	Not sampled	Not sampled	***+*** *Bu*. *truncatus* (1/1) *Bu*. *globosus* (0/1) ***+*** *Bu*. *senegalensis* (1/1)

As for rodents, schistosomes infection in snails was highly focal. No positive molluscs were found in zone A, where positive rodents were found. All positive molluscs were found in 4 transmission sites of zone B, positive rodents being found in two of them. Infected *Bi*. *pfeifferi* were repeatedly found in Guéo with a local prevalence reaching 18.9% in April 2021 and only once in Gankette Guinth and Gankette Balla with a local prevalence of 12.8% and 9.1% respectively, in August 2022 (Table 5). Infected *Bu*. *truncatus* were found in two transmission sites (Gankette Balla and Takh) and *Bu*. *senegalensis* in a unique site (Takh) with a local prevalence ranging between 14.3% and 100% (Table 5).

### Molecular characterisation of schistosomes from rodents and snails

Amongst all adult schistosomes collected from rodents (n = 191) *cox1* RD-PCR revealed the presence of 188 specimens assigned to *S*. *mansoni* (98.4%), one female worm to *S*. *bovis* and one male worm to *S*. *haematobium*, while one remaining male worm could not be identified (S3 File). All randomly selected *cox1* PCR amplicons sequenced (n = 17) confirmed profiles obtain from the RD-PCR except one unreadable sequence (S3 File). Sequencing of the ITS gene gave reliable data for 181 sequences while 10 sequences (all assigned to *S*. *mansoni cox1*) could not be interpreted due to bad quality or contaminations (S3 File). However, all individuals previously assigned to a species based on *cox1* had a congruent ITS sequence and none of the specimens examined showed polymorphism within the ITS sequence suggestive of hybridization (Tables 6 and 7).

**Table 6:** Polymorphic positions identified within *S*. *mansoni* ITS gene (925 bp). Ref = reference sequence with GenBank accession.

Polymorphic positions	29	51	89	216	251	387	874	925	Species	Nb. of sequences
**Ref (AF531312)**	A	T	G	T	T	C	C	A	*Schistosoma rodhaini*	**-**
**Ref (AY446082)**	A	T	A	C	T	C	T	A	*Schistosoma mansoni*	**-**
**Variant_Sm1**	A	T	A	C	T	C	T	A		219/316 (69.3%)
**Variant_Sm 2**	A	T	A	C	T	C	T	A/T		65/316 (20.6%)
**Variant_Sm 3**	A	C	A	C	T	C	T	A		14/316 (4.4%)
**Variant_Sm 4**	A	T	A	C	T	C	T	T		7/316 (2.2%)
**Variant_Sm 5**	A	T	A	C	C	C	T	A		3/316 (0.9%)
**Variant_Sm 6**	G	T	A	C	T	C	T	A		2/316 (0.6%)
**Variant_Sm 7**	A	C	A	C	T	C	T	A/T		2/316 (0.6%)
**Variant_Sm 8**	A	T	A	C	T	T	T	T		1/316 (0.3%)
**Variant_Sm 9**	T	C	A	C	T	C	T	A		1/316 (0.3%)
**Variant_Sm 10**	A	T	A	C	C	C	T	A/T		1/316 (0.3%)
**Variant_Sm 11**	A	T	A	C	T	T	T	A/T		1/316 (0.3%)

**Table 7:** Polymorphic positions identified within the ITS gene (932 bp) between *S*. *haematobium* and *S*. *bovis*. Ref = reference sequence with GenBank accession.

Polymorphic positions	70	380	553	722	777	827	897	930	Species	Nb. of sequences
**Ref (MT884914)**	A	A	C	G	C	G	C	A	*Schistosoma haematobium*	*-*
**Ref (MT580950)**	G	A	C	A	T	A	T	A	*Schistosoma bovis*	*-*
**Ref (OX103898)**	G	A	C	A	T	A	T	A	*Schistosoma guineensis*	*-*
**Ref (MT580947)**	A	A	C	A	T	A	T	A	*Schistosoma curassoni*	*-*
**Variant_Sb1**	G	A	C	A	T	A	T	A	*Schistosoma bovis*	6/11 (54.5%)
**Variant_Sb2**	G	A	C	A	T	A	T	A/T		5/11 (45.5%)
**Variant_Sh1**	A	A	C/T	G	C	G	C	A	*Schistosoma haematobium*	5/8 (62.5%)
**Variant_Sh2**	A	A	C/T	G	C	G	C	A/T		2/8 (25.0%)
**Variant_Sh3**	A	A/G	C/T	G	C	G	C	A/T		1/8 (12.5%)

A total of 69 miracidia collected from rodents (n = 11) were subject to *cox1* RD-PCR which confirmed the presence of *S*. *mansoni* in all of those infected individuals except one individual (where an unidentified adult male was previously found) with miracidia assigned to *S*. *haematobium* (n = 3 miracidia). These results were further confirmed by *cox1* sequencing (n = 10 miracidia). Sequencing of the ITS gene gave reliable results for 49 miracidia while 17 sequences could not be interpreted and 3 were not sequenced. No hybrid profile was found amongst the miracidia analysed.

These findings show the predominance of *S*. *mansoni* in rodents (16/18) through space and time but also the presence of *S*. *haematobium* in two *M*. *huberti* from Temeye Lewa. As shown from the presence of hatched miracidia, *S*. *haematobium*, which is usually restricted to humans, seems viable in rodents. Another interesting discovery was the presence of a *S*. *bovis* female paired with a male *S*. *mansoni* in one *M*. *huberti* from Temeye Soubalo. Unfortunately, as no eggs or miracida could be collected we could not evidence the presence of potential first-generation hybrids between these two species.

Finally, over the 29 snails shedding schistosomes, 123 cercariae were analysed by *cox1* RD-PCR. All cercariae (n = 103) emitted from *Bi*. *pfeifferi* (n = 25) were identified as *S*. *mansoni*, whereas cercariae (n = 18) shed from *Bu*. *truncatus* (n = 3) and *Bu*. *senegalensis* (n = 1) were either assigned to *S*. *bovis* (n = 16) or *S*. *haematobium* (n = 2). One of the *Bu*. *truncatus* was found infected with both *S*. *bovis* (n = 2 cercariae) and *S*. *haematobium* (n = 2 cercariae). The sequencing of 18 *cox1* amplicons confirmed profiles obtained from the RD-PCR. Sequencing of the ITS gene gave reliable results for 105 cercariae while 14 could not be interpreted and 4 were not sequenced. All *S*. *mansoni cox1* profiles were congruent with the ITS sequences, thus no hybrids with this species were detected. However, although cercariae from two *Bu*. *truncatus* and one *Bu*. *senegalensis* had congruent results between *cox1* and ITS profiles indicative of a *S*. *bovis* infection, one remaining *Bu*. *truncatus* was infected with both *S*. *haematobium* and S. *haematobium x S*. *bovis* hybrids (discordance between *S*. *bovis cox1* and *S*. *haematobium* ITS within the same individual). All molecular data at the individual parasite’s level is available in S3 File.

Overall, we obtained 335 ITS gene sequences and 44 *cox1* sequences from schistosomes recovered form rodents and snails in the vicinity of Lake Guiers, Senegal. The majority of ITS sequences were assigned to *S*. *mansoni* (316 sequences: 179 adult worms, 47 miracidia and 90 cercariae) with 11 variants (Table 6). Double peaks were found in only one variant at position 925 and did not refer to *Schistosoma* species or known hybrids.

The remaining ITS sequences belong either to *S*. *bovis* (11 sequences with 2 variants) or *S*. *haematobium* (8 sequences with 3 variants). No double peaks were observed in the chromatograms at the five polymorphic positions (70, 722, 777, 827, and 897) between *S*. *haematobium*, *S*. *bovis* or *S*. *curassoni (*Table 7). However, double peaks were found at other unknown positions (380, 553, and 930) and did not refer to *Schistosoma* hybrids.

## Discussion

The involvement of rodents in the transmission of schistosomiasis has raised concerns on the significant threat they may pose to the disease control efforts across Africa [12,20,26,27,29,31–33,53,58,84]. The role of vertebrate hosts other than humans has been largely overlooked in control strategies. Very little is known on the spatio-temporal variations of infection at the animal-parasite interface, a parameter that could help adapting the management of the disease. Based on a two-year survey around Lake Guiers, in northwestern Senegal, our study provides insight on the structure and population dynamics of small mammal communities as well as on their spatial and temporal variations of infection with schistosomes in this area.

The diversity of small mammal species was relatively low at transmission sites. Only four species were caught with overall capture rates were similar to those previously recorded in the area [26,27]. Very low capture rates were expected for *T*. *pygargus* living in dry habitats [63], however they were more surprising for *C*. *viaria* that is quite broadly distributed [85,86] including in humid habitats of the Senegal River Basin. As expected, the dominant species was *M*. *huberti*, known to inhabit humid and partially flooded environments in this regions [87]. Overall, small mammal capture rates varied between sampling periods, with lowest values observed during the rainy season. This observation, especially significant in *M*. *huberti* whose preferred habitat was systematically well sampled during all sampling periods, reflects lower rodent population sizes usually recorded in Sahelian environments at that period due to population turn over [28,63]. However, we cannot exclude that repeated sampling over time may have influenced small mammal abundance locally. For *A*. *niloticus*, the significant decline in capture rates after the first sampling period probably results from a lesser trapping effort after April 2021 along crop fields and gardens, which are the preferred habitats of this species [63], as we focused on habitats close to water bodies to favour the capture of rodents infested by schistosomes. At the spatial scale, *M*. *huberti* was dominant in all localities except one (Mbane where various gardens were sampled) and there was a higher diversity of species in zone A, whilst only *M*. *huberti* and *A*. *niloticus* were trapped in zone B. A possible reason for this may be the higher diversity of habitats encountered in zone A, including humid riparian habitats boarding the lake, various crop fields and gardens, irrigation canals and livestock watering sites, compared to more homogeneous habitats along the lake in zone B. Concerning rodent population structure, there were no significant differences of sex ratio between sampling periods or geographical zones, within rodent species. On the contrary, the higher proportion of juveniles during the first semester (especially in February-March 2022), is congruent with the known pattern of reproduction of these rodents in Senegal [63,87,88]. The unusually high proportion of juveniles recorded for *A*. *niloticus* in August 2022 (zone A) may result from an exceptional reproductive event linked with local climatic or environmental conditions [87].

The prevalence of schistosomes in small mammal communities was relatively low on a global scale (3.6%), consistent with findings from other studies across Africa [26,27,29,31], in contrast to *foci* in the Caribbean [17,19], Southern America [18] or Asia [89,90]. However, *Schistosoma* infections seem to strongly depend on the spatio-temporal scale at which it is inferred from and on the host and parasites species considered [53]. As previously reported, both *A*. *niloticus* and *M*. *huberti* were found infected with schistosomes along Lake Guiers [26,27,29]. *Mastomys huberti* appeared as the main reservoir for schistosomes in this area as only one *A*. *niloticus* was found infected. Infected *A*. *niloticus* in Senegal were previously mainly found in the surroundings of Richard-Toll and Djidiery [26,27,29]. This observation and our results suggest local specificities driving the transmission of schistosomes in different rodent species. It is probable that differences in habitats found around Richard-Toll (i.e., large networks of crop fields and irrigation canals for the cultivation of rice and sugarcane, that are suitable for *A*. *niloticus*), and rodent species ecology, could at least in part, be at play. The use of different habitats and their proximity to various freshwater sources could explain the higher prevalence rates of schistosomes in *M*. *huberti* compared to *A*. *niloticus* occupying drier habitats in the Lake Guiers area (see also [63,87]). This therefore suggests the existence of contrasting epidemiological situations that may arise even at small spatio-temporal scales necessitating to assess *Schistosoma* infections in animals at a fine scale.

Infected rodents were found across all sampling periods, with no significant difference in prevalence or intensity of infection over time. However, most parasitized rodents and schistosomes were retrieved during or just after the rainy season, which coincides with the dominance of older individuals in rodent populations. Previous studies conducted in Richard-Toll also showed similar temporal variations in infection with a prevalence peak in July and November but no clear seasonal pattern over the years [28]. Nonetheless, the cumulative evidence of schistosomes found mostly, if not exclusively, in adult rodent individuals (this work, but also [28,29] and [26,27]) is not surprising. Indeed, rodents have a short lifespan and new-born individuals remain nested until two to three weeks of age [91]. Even if rodents rapidly come in contact with contaminated water sources, the development of mature adult worms that produce eggs and contribute to transmission takes several weeks (~5–7 weeks post-exposure). Therefore, it is unlikely to detect infected rodents less than two months old using current field methodologies (perfusion and dissections, coprology, miracidia hatching). Likewise, the rapid turnover in rodent populations may impose that the window of detection and transmission of the parasites from such hosts may be relatively short. The population dynamics and age structure of hosts thus appear as important parameters that can influence *Schistosoma* infections through time and space. Focusing on adult individuals of at least 3–4 months of age could be beneficial for future field studies conducted on rodents. Further research and the development of new diagnostic methods and tools for animals easily employable in the field are needed in order to gain a deeper understanding of *Schistosoma* temporal transmission dynamics in small mammals, especially in relation to human reinfection after MDA of PZQ.

The distribution of infected rodents in the study area was highly focalised, a common feature of schistosomiasis transmission. Most sites where infected rodents were found and highest prevalence levels were observed along irrigation canals in zone A compared to sites with a direct access to the lake in zone B. This suggests that irrigation canals may be important sites for *Schistosoma* infections in both animals and human. Studies on schistosomiasis in human have indeed indicated that irrigation canals serve as hotspots for transmission despite MDA, likely due to factors such as proximity to households, ease in water accessibility, frequency of domestic and recreational activities and also a higher density of intermediate hosts snail populations [92]. It is important to note that although the prevalence in rodents could be high locally and temporally (reaching up to 100% in certain localities and at particular sampling periods), the hight variability of infection intensity at the individual host level highlights the dynamic role that rodents may play in schistosomes transmission. Therefore, both the prevalence and intensity of infection needs to be assessed to define the transmission potential of a particular host within a specific spatio-temporal context. Overall, our results indicate recurrent transmission of schistosomes in rodents at particular sites throughout several months (7–8 months) and more variation in *Schistosoma* infections across space than over time.

Recent records from villages along the Lac Guiers and the Senegal River basin show that in human populations *S*. *mansoni* is substantially prevalent (up to 45%) [27,45], but urogenital schistosomiasis is the most widespread (88% prevalence in children of which 72% where infected with *S*. *haematobium–S*. *bovis* hybrids) [45]. Likewise, the prevalence of schistosomes in sympatric livestock populations is extremely high (up to 94% for *S*. *bovis* in cattle). Interestingly, in rodents the most widespread schistosome species found was *S*. *mansoni*, confirming their role as true reservoir hosts in the global burden of intestinal schistosomiasis in that region [26,27,29]. Additionally, other schistosome species of human (*S*. *haematobium*) and animal (*S*. *bovis*) significance were sporadically found in rodent from Temeye. While *S*. *bovis* has been found in several wild rodents in sub-Saharan Africa [24,26,27,31,39,40], *S*. *haematobium* is considered as highly specific to humans [5,31,36]. The finding of two *M*. *huberti* infected with single male *S*. *haematobium*, including one with viable miracidia, suggests that *M*. *huberti* could be a spill-over host that may, to some extent, play a role in the transmission of urogenital schistosomiasis in Senegal. It also supports the need for more sensitive diagnostic tools for animal hosts, including detecting single sex infections that can easily pass unnoticed [93]. The presence of a heterospecific pair between a female *S*. *bovis* and a male *S*. *mansoni* in one *M*. *huberti* from Temeye was another notable finding. Rodents have been claimed as “biotic hubs” in which various *Schistosoma* species can meet and potentially hybridize [26,31,53]. For instance, Catalano et al. 2018 [26] previously reported a pair composed of a *S*. *mansoni* male with a hybrid female (ITS: S. *haematobium* x COI: *S*. *bovis*) in *M*. *huberti* from the same village, indicating that Temeye, may be a significant location for interactions between schistosome species. In our particular case, the female of the *S*. *bovis* x *S*. *mansoni* pair was immature (visual observation) and since the rodent infection was likely recent, no miracidia could be collected to confirm potential hybrids. Although these hybrids do not occur naturally, laboratory experiments have shown that *S*. *mansoni* and *S*. *bovis* can pair, producing parthenogenetic offspring that infect the maternal species’ intermediate snail hosts [94]. This indicates that co-infection and heterospecific pairs in reservoir hosts like rodents, while not leading to hybridization *per se*, may still enable maternal species transmission without conspecific mates. The role of rodents as biotic hubs requires further clarification, as it has strong implications for the parasites transmission and control [26,38,59]. In the same vein, although *S*. *haematobium’s* capacity to infect rodents could have been due to genetic introgression from *S*. *bovis*, no such hybrids were found in rodents. The low prevalence of *S*. *bovis* and *S*. *haematobium* and the absence of hybrids in the rodent populations suggest behavioural divergence in local host-parasite interactions. However, this also highlights diverse epidemiological situations, where different animal reservoirs could locally play a role in parasite transmission. For instance, in Benin, *M*. *natalensis* and *R*. *rattus*, two rodent species absent in Northern Senegal, were found infected with *S*. *bovis* x *S*. *haematobium* hybrids [53]. This difference in local hosts-parasites interactions may be influenced by the cercarial emergence rhythm from the intermediate snail hosts, known as chronobiological polymorphism [95]. Indeed, *S*. *bovis* and *S*. *haematobium* typically follow a strictly diurnal rhythm, with cercarial emission peaking in the early morning and mid-day, respectively. However, *S*. *bovis* x *S*. *haematobium* hybrids in Benin exhibit atypical chronobiological patterns (early diurnal, late diurnal, nocturnal, double-peak emission patterns) allowing them to exploit a diversity of local hosts, including cattle, rodents and humans [49,53]. In Senegal, the chronobiological polymorphism of *Schistosoma* species, including hybrids needs to be assessed, especially since the presence of *S*. *mansoni* in both humans and the mainly nocturnal rodent *M*. *huberti* [96] could indicate the existence of various chronotypes [95].

Finally, we found *Bi*. *pfeifferi*, *Bu*. *truncatus*, and *Bu*. *senegalensis* shedding schistosomes near small mammals capture sites. Most infections occurred in *Bi*. *pfeifferi*, all transmitting *S*. *mansoni*, predominantly when snail populations peak in spring and early summer [97]. This is potentially congruent with a subsequent higher number of *S*. *mansoni* infected rodents in August and November, although our non-standardized opportunistic sampling did not allow us to compare snail infection levels through space and time. Infected *Bulinus* sp. were rarer with *Bu*. *truncatus* transmitting either *S*. *bovis* or *S*. *haematobium* and S. *haematobium x S*. *bovis* hybrids. We also provide further confirmation for *Bu*. *senegalensis* transmitting *S*. *bovis* in the area [98]. Positive snails were found in only 4 sites of zone B, overlapping with positive rodents in 2 sites. No positive molluscs were recovered in zone A, where positive rodents were caught. The inconsistency between snail and rodent infection can be attributed to several factors. First, the focality of transmission, along with the typically low prevalence of snail populations and our opportunistic sampling, likely led to missing infected snails. Second, the detection of infected rodents suggests that snails were shedding schistosomes several weeks earlier, meaning a temporal gap between hosts that could contribute to the observed patterns. Finally, the transmission at particular site of *S*. *bovis* and *S*. *haematobium* that seem to be rarer in rodents compared to *S*. *mansoni*, could further explain the absence of infected rodents in certain sites. Therefore, the absence of infected snails does not necessarily rule out ongoing transmission, while finding infected snails confirms active schistosomiasis transmission even if rodents are not infected. Assessing infections in definitive and intermediate hosts may provide complementary insights into schistosomiasis spatial and temporal occurrence.

In conclusion, our study emphasizes the role of rodents in the transmission of African schistosomes by acting as reservoirs for *S*. *mansoni* or spill-over hosts for *S*. *bovis* and *S*. *haematobium*. It highlights the fine spatio-temporal variations of infections in small mammal communities found in the vicinity of Lake Guiers in northern Senegal. Overall, infections were found predominantly during or just after the rainy season, a time when older individuals dominate in rodent populations. Variations in *Schistosoma* infections in rodents seemed to be greater over space than time, indicating the need for a better understanding of the parasite’s epidemiology at various transmission sites. Our work also emphasises the role of rodents as biotic hubs allowing various *Schistosoma* species to encounter and potentially hybridize. The focality of transmission, the high variability in infection parameters of rodents and molluscs that sometimes appear incongruent, and the relatively low number of infected rodents caught, make it difficult to drawn general patterns usable to adapt control strategies. New methods and tools enabling an easier monitoring of *Schistosoma* infection in animals would enable to conduct larger spatio-temporal surveys. Combined with fine scale population genomic tools, they should provide unprecedented resolution on schistosomes zoonotic transmission, parasite diversity and their evolutionary relationships within and between humans, animals and the environment, allowing adapting local measures for a sustained control of the disease.

## Supporting information

S1 Table Summary of the most parsimonious Generalized Linear Mixed Models (GLMMs) finally selected. AICc: Akaike’s information criterion corrected for finite sample size. Δ: difference between the model selected and the model with the lowest AICc. LRT: Likelihood-ratio test.
(DOCX)

S1 File Small mammal captures information over time and across space. (XLSX)

S2 File Autopsy data from trapped small mammals over time and across space. (XLSX)

S3 File Molecular data of adult worms, miracidia and cercariae found over time and across space in rodents and snails. (XLSX)
