# Supplementary material for: African schistosomes in small mammal communities: Perspectives from a spatio-temporal survey in the vicinity of Lake Guiers, Senegal
Source: PLoS Negl Trop Dis. 2024 Dec 23;18(12):e0012721. doi: 10.1371/journal.pntd.0012721 (PMC11706494; doi:10.1371/journal.pntd.0012721)
Supplement: S1 Table — AICc: Akaike’s information criterion corrected for finite sample size. Δ: difference between the model selected and the model with the lowest AICc. LRT: Likelihood-ratio test. (DOCX) [file pntd.0012721.s001.docx]

**S1 Table**: Summary of the most parsimonious Generalized Linear Mixed Models (GLMMs) finally selected. AICc: Akaike’s information criterion corrected for finite sample size. Δ: difference between the model selected and the model with the lowest AICc. LRT: Likelihood-ratio test.

| **Dataset considered** | **Response variable** | **AICc (Δ)** | **Predictors selected** | **LRT** | **p-value** |
| --- | --- | --- | --- | --- | --- |
| All species | Capture | 3031.1 (0) | Sampling period | 28.014 | 3.607e-06 |
|  |  |  | Village | 18.241 | 0.01093 |
| *Arvicanthis niloticus* | Capture | 936.7 (0) | Sampling period | 18.238 | 0.0003928 |
|  |  |  | Village | 20.884 | 0.0039460 |
|  | Sex | - | None | - | - |
|  | Age | 125 (0.46) | Sampling period | 18.552 | 0.0003383 |
| *Mastomys huberti* | Capture | 2318.6 (0) | Sampling period | 16.526 | 0.0008846 |
|  |  |  | Village | 25.376 | 0.0006508 |
|  | Sex | - | None | - | - |
|  | Age | 292.2 (0.58) | Sampling period | 13.026 | 0.004581 |
|  |  |  | Village | 17.637 | 0.013718 |
